# An unstructured proteasome inhibitor comes into focus

**DOI:** 10.1016/j.jbc.2023.105145

**Published:** 2023-08-09

**Authors:** Antonia A. Nemec, Robert J. Tomko

**Affiliations:** Department of Biomedical Sciences, Florida State University College of Medicine, Tallahassee, Florida, USA

## Abstract

The inhibitory mechanism of an intrinsically disordered proteasome inhibitor identified over 30 years ago has finally been revealed by cryo-electron microscopy by Hsu *et al.* in a recent report in the *Journal of Biological Chemistry*. The structure, coupled with biochemical and cell-based experiments, resolves lingering questions about how the inhibitor achieves multisite inhibition of proteasomal protease activity, while raising several exciting new questions on the nature of proteasome subpopulations in the process.

Weighing in at 2.5 MDa and containing 66 individual protein subunits, the 26S proteasome is the largest and most complicated protease known to science (1). The proteasome destroys damaged, defective, and unneeded proteins using a combination of trypsin-like, chymotrypsin-like, and caspase-like peptidase activities. These activities are mediated by three distinct subunits, each present in two copies, within the central barrel-shaped core of the proteasome (Fig. 1). Given that alterations to proteasomal proteolysis underlie several human diseases, there has been tremendous interest in the development of proteasome inhibitors as therapeutics. Small-molecule inhibitors of the proteasome are currently first-line therapies for the treatment of certain cancers and are in various stages of development for certain autoimmune disorders and infectious diseases, among other conditions.

**Figure 1:**
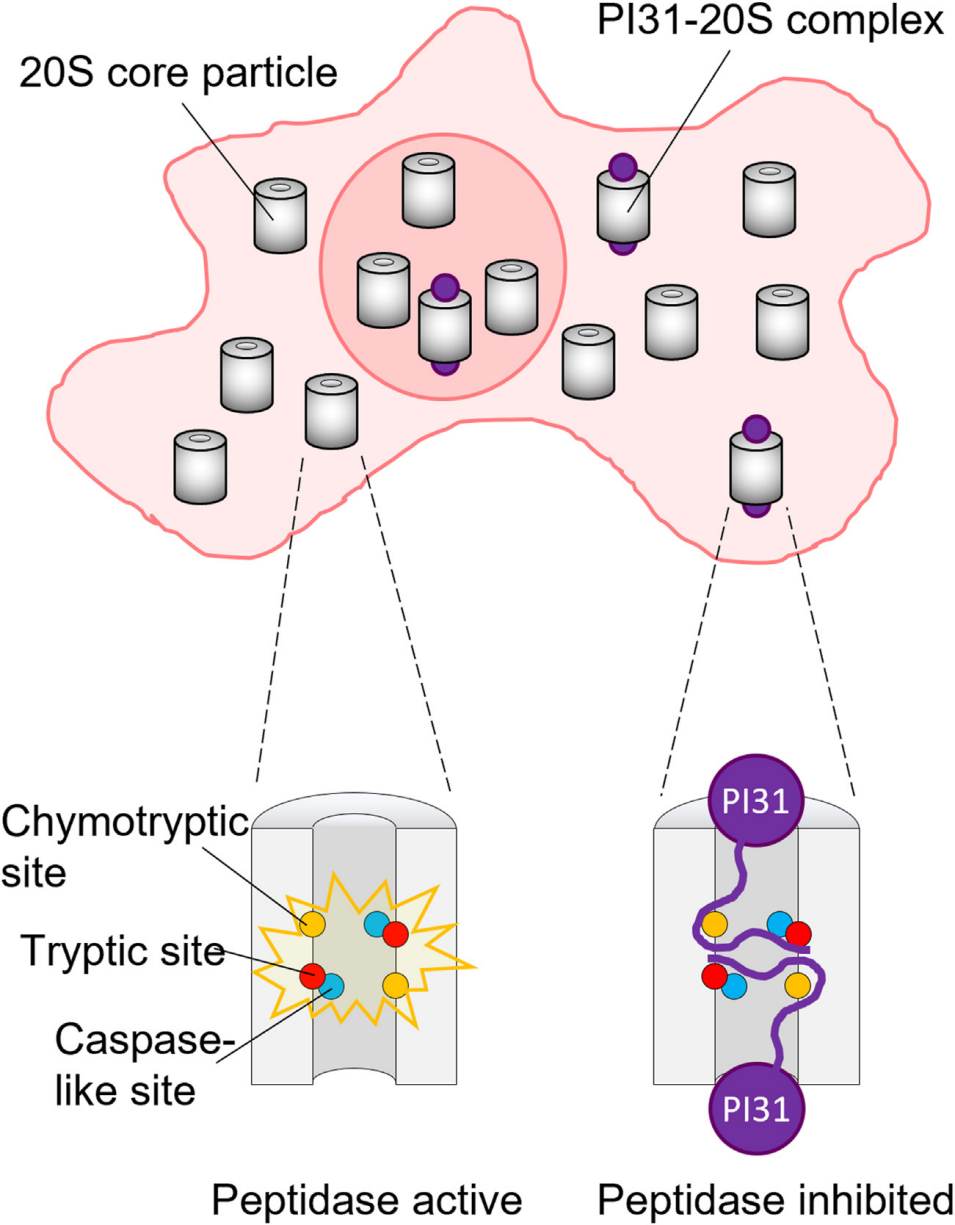
**Proteasomal 20S core particles in a mammalian cell are shown either as free complexes (*gray cylinders*) or with PI31 (*purple spheres*) bound.** The caspase-like, trypsin-like, and chymotrypsin-like sites of the core particle are shown in *blue*, *red*, and *orange*, respectively. PI31 docks over the central pore of the core particle, and its proline- and glycine-rich C-terminal domain snakes from active site to active site, impairing proteolysis. Other regulators of the 20S core particle that exist in cells are omitted from the figure for simplicity. PI31, Proteasome Inhibitor of 31,000 Da.

Although the proteasome was originally thought of as a bland, unchanging “garbage disposal,” several early studies hinted that it was a target of surprisingly complex regulation inside cells. In the early 1990s, the DeMartino group (2, 3, 4) identified several protein regulators of the proteasome by screening biochemical fractions of erythrocyte cell extracts for impairment or enhancement of proteasomal substrate degradation. Among the hits was a proline- and glycine-rich protein that became known as Proteasome Inhibitor of 31,000 Da (PI31). PI31 was shown by the DeMartino group, and later others, to potently inhibit all three types of peptidase sites within the proteasome (Fig. 1). Despite being a protein itself, it somehow completely evaded being degraded in the process. Over the next 30 years, several groups have attempted to decipher how PI31 could inhibit the two sets of three distinct enzymatic sites present in separate locations within the core of the proteasome, and to understand PI31’s physiological role(s). However, many studies yielded results seemingly inconsistent with PI31 being a proteasome inhibitor in cells, further muddying the waters.

A new study from the Li and DeMartino groups (5) is one of three articles published in short succession that provide a detailed picture of how PI31 inhibits the proteasome (6, 7). In the current article, the authors determined the cryo-electron microscopy structure of the PI31-bound core of the proteasome, called the 20S core particle (CP), at 2.5 Å resolution. This high-resolution structure yielded a near-atomic picture of how PI31 inhibits the three distinct peptidase sites found within the CP, which the authors validated with mutagenesis. They discovered that PI31 docks its N-terminal dimerizing region, known as an FP domain, onto the flat surface of the barrel-shaped CP. The long unstructured C-terminal proline- and glycine-rich domain of PI31 then inserts into the central pore of the CP barrel through which substrates would normally access the peptidase active sites. In the interior, this unstructured C-terminal domain of PI31 snakes from peptidase site to peptidase site within the CP structure, trapping key catalytic residues of the peptidase subunits in nonproductive states.

The interaction of PI31 with the individual peptidase active sites revealed several of the strategies that this inhibitor uses to block proteolysis. All three types of peptidase sites within the proteasome utilize a threonine side chain hydroxyl group as the nucleophile that initiates attack on the peptide bond; this threonine hydroxyl is thought to be deprotonated for catalysis by a nearby lysine residue. PI31 forms hydrogen bonds *via* conserved aspartate residues with the catalytic threonines of the trypsin-like and chymotrypsin-like sites, which likely prevents the deprotonation necessary for nucleophilic attack. Although inhibition of the caspase-like site was not directly visualized in this structure, biochemical experiments and insights from other studies suggest that a similar mechanism is used to inhibit this activity (6, 7). Closer examination of the interaction of PI31 with the trypsin-like and chymotrypsin-like sites illuminates additional features that allow PI31 to escape degradation. The docking of PI31 into the trypsin-like site of the proteasome positions a proline as the C-terminal residue in what would otherwise be the scissile bond; indeed, the presence of a proline at this critical position often prevents cleavage by trypsin (8) and likely also renders PI31 resistant to cleavage by the trypsin-like activity of the proteasome. In contrast, PI31 docks into the chymotrypsin-like pocket in the reverse orientation of how a typical substrate would dock, mispositioning any peptide bonds relative to the threonine nucleophile. This was particularly surprising because yeast PI31 was previously reported to dock into the chymotrypsin-like site in the conventional orientation assumed by a substrate (6).

In addition to these insights, the Li and DeMartino groups demystified two additional questions surrounding PI31 function. First, PI31 is known to form homodimers *via* its N-terminal FP domain (9); is the active form of PI31 a dimer or a monomer? Using mutagenesis of the dimerization interface, the authors demonstrated that PI31 retained essentially full inhibitory activity when homodimerization was impaired. Second, although PI31 was generally agreed to function as a proteasome inhibitor *in vitro*, several groups had failed to demonstrate such an inhibitory effect in cells. These findings came with the caveat that PI31 may not have been expressed highly enough to inhibit a significant fraction of the total cellular proteasome population, yielding a false-negative result. By carefully titrating the expression levels of PI31, the authors demonstrated that at sufficient abundance, PI31 does inhibit proteasomes in cells as observed *in vitro*, thereby rationalizing the unexpected lack of inhibition observed in previous studies.

Although the study by the Li and DeMartino groups has solved several key questions regarding PI31, it also raises several additional questions regarding the cellular function(s) of this highly conserved proteasome inhibitor. For example, given that PI31 is highly substoichiometric to proteasomes in cells, it suggests that PI31 functions to regulate the activity of only a subpopulation of the total proteasome pool. However, the nature and distinguishing features of this subpopulation of proteasomes is completely unknown, and no physiological or pathological conditions that enhance expression of PI31 are currently known. Although most CP in the cell is capped at one or both ends of the barrel by regulatory complexes, PI31 may serve to inhibit the small population of free CP. Notably, PI31 has also been reported to promote trafficking of proteasomes *via* interaction with dynein motors (10); the inhibitory function of PI31 may serve to restrict the proteolytic activity of proteasomes in transit until they reach their cellular destinations. Although its physiological role(s) remain incompletely understood, the current work provides an outstanding framework to guide studies of how PI31-dependent proteasome inhibition regulates cellular functions.

## Conflict of interest

The authors declare that they have no conflicts of interest with the contents of this article.
